# Colostrum Feeding among Newborns Visiting the Outpatient Department of Pediatrics of a Tertiary Care Centre: A Descriptive Cross-sectional Study

**DOI:** 10.31729/jnma.8062

**Published:** 2023-03-31

**Authors:** Sharda Acharya, Bibechan Thapa, Ajaya Kumar Dhakal, Saurav Kumar Singh

**Affiliations:** 1 Department of Pediatrics, KIST Medical College and Teaching Hospital, Mahalaxmi, Lalitpur, Nepal; 2 KIST Medical College and Teaching Hospital, Mahalaxmi, Lalitpur, Nepal

**Keywords:** *colostrum*, *exclusive breastfeeding*, *newborns*, *prevalence*

## Abstract

**Introduction:**

Colostrum is the thick yellowish breast milk that is produced during the first 3-5 days of childbirth. Feeding colostrum protects the newborn from various diseases, thus promoting the overall well-being of the newborn. The objective of this study was to find out the prevalence of colostrum feeding among newborns visiting the Department of Pediatrics in a tertiary care centre.

**Methods:**

A descriptive cross-sectional study was done among infants presenting to the Department of Pediatrics in a tertiary care centre. Ethical approval was taken from the Institutional Review Committee (Reference number: 2078/079/107). The duration of study was six month from 12 February 2022 to 12 August 2022. A pre-designed questionnaire was used for face-to-face interviews. Convenience sampling was done. Point estimate and 95% Confidence Interval were calculated.

**Results:**

Among 350 newborns, colostrum was fed to 305 (87.14%) (83.63-90.65, 95% Confidence Interval) newborns. A total of 180 (59.02%) were breastfed within 1 hour of delivery.

**Conclusions:**

The prevalence of colostrum feeding was higher in our study than in other studies done in similar settings.

## INTRODUCTION

Colostrum is the first milk that is very important for newborns in protecting against infections since it is rich in immunoglobin G.^1,2^ Various bacterial, viral, fungal and protozoal infections of the neonates can be reduced by feeding colostrum.^3^ Optimal breastfeeding practices, reflected by early initiation and feeding of colostrum, avoidance of prelacteal feeds, and continued exclusivity or predominance of breastfeeding, are critical for assuring proper infant nutrition, growth and development.^4,5^

Despite many global efforts being made, the practices of discarding colostrum and the early use of commercial milk formula are still prevalent. Lack of social support, poverty, disability, and perinatal complications could be some of the reasons for shorter breastfeeding duration and discarding of colostrum.^6^ There have different studies that show that children not feeding on colostrum are more at risk of developing infections, stunting underweight and wasting.^1^

The objective of this study was to find out the prevalence of colostrum feeding among infants visiting the Department of Pediatrics in a tertiary care centre.

## METHODS

A descriptive cross-sectional study was conducted in the Outpatient Department of Pediatric of KIST Medical College and Teaching Hospital, Lalitpur, Nepal after receiving ethical approval from the Institutional Review Committee (Reference number: 2078/079/107). The duration of study was six month from 12 February 2022 to 12 August 2022. Infants less than 6 months of age were enrolled in the study. Mothers of twin/multiple infants were excluded from the study as there could be chances of inadequate breast milk production to fulfill the needs of two infants by a single mother. Apart from these mothers having chronic medical illnesses (active tuberculosis, leprosy, and AIDS) and conditions in which breastfeeding is contraindicated were also excluded. Infants with specific feeding problems (cleft lip or palate, congenital heart disease, severe illness during the neonatal period, or delayed developmental milestones) were also excluded. Convenience sampling was done. The sample size was calculated using the following formula:


$$\begin{array}{ll} \text{n} & ={\text{Z}}^{2}\times \frac{\text{p}\times \text{q}}{{\text{e}}^{\text{2}}}\\ & =1.96^{2}\times \frac{0.835\times 0.165}{0.04^{2}}\\ & =331 \end{array}$$

Where,

n = minimum required sample size
Z = 1.96 at 95% Confidence Interval (CI)
p = prevalence of colostrum feeding taken from the previous study, 83.5%^4^
q = 1-p
e = margin of error, 4%

The calculated minimum required sample size was 331. However, we enrolled 350 infants in our study.

A pre-designed questionnaire was administered directly to the participants by the investigator via face-to-face interviews.^7^ Those infants that were fed with colostrum (thick yellowish breastmilk) were taken as our required criteria.^2^ Questionnaires were created in the Nepali language to optimize proper communication. Investigators read out questions written in the questionnaire and answers were marked by the investigator as per the reply of the participants. After completion of all the questions, all the responses given by the participant himself/herself were read out to the participants to ensure the recorded responses were correct.

Data were entered and analyzed using IBM SPSS Statistics version 26.0. Point estimate and 95% Confidence Interval were calculated.

## RESULTS

**Figure 1:**
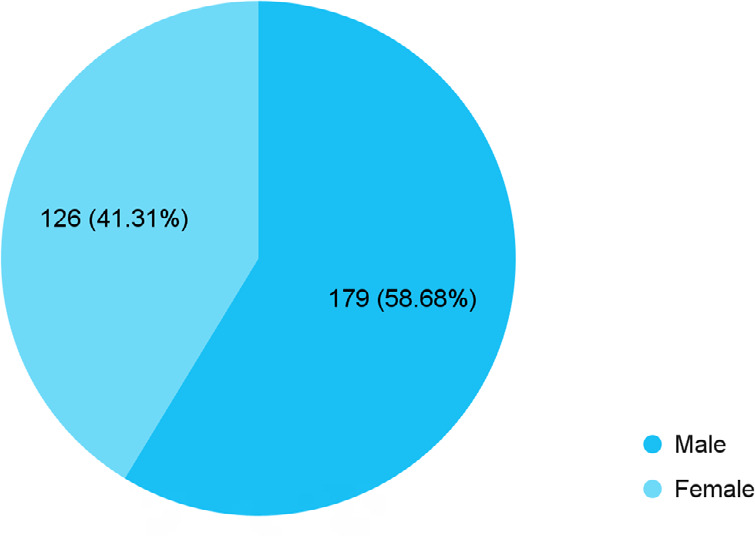
Gender-wise distribution of infants with colostrum feeding (n= 305).

Among 350 infants, 305 (87.14%) (83.63-90.65, 95% CI) were fed with colostrum. Out of them, 179 (58.69%) were male (Figure 1).

Among mother who fed colostrum, 243 (79.67%) followed Hinduism religion, 42 (13.77%) followed Buddhism (Table 1).

**Table 1:** Religion-wise distribution (n = 305).

Religion	n (%)
Hinduism	243 (79.67)
Buddhism	42 (13.77)
Christianity	15 (4.92)
Islam	3 (0.98)
Others	2 (0.66)

**Figure 2:**
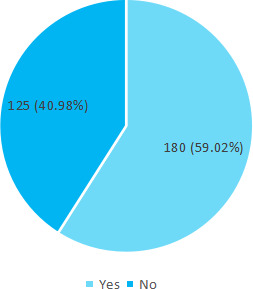
Breastfeeding within 1 hour (n= 305).

A total of 180 (59.02%) were breastfed within 1 hour of delivery (Figure 2).

Around 179 (58.69%) infants were breastfed 8 to 12 times in 24 hours while 7 (2.30%) of the infants were breastfed as per the demand of the infant (Table 2).

**Table 2:** Breastfeeding frequency (n= 305).

Frequency	n (%)
<4 times	12 (3.93)
4-8 times	76 (24.92)
8-12 times	179 (58.69)
>12 times	31 (10.16)
As demand	7 (2.30)

The birth weight of 262 (85.9%) infants was between 2500-4000 grams (Table 3).

**Table 3:** Birthweight of infants (n=305).

Birthweight (grams)	n (%)
<2500	37 (12.13)
2500-4000	262 (85.9)
>4500	6 (1.97)

## DISCUSSION

This study revealed that 87.14% of infants were fed colostrum after birth which was slightly higher (83.5%) than a nation-wide study done in Nepal.^4^ In a similar study done in lowlands of Nepal, only 67% of infants were given colostrum.^8^

In our study 59.02% of infants were breastfed within one hour of delivery similar to the data from studies done in different parts of Nepal.^7,9,10^ But it is more as compared to some of the nation-based studies.^4,11^ In a similar study done in an urban population of Western Nepal, Midwestern Nepal and Eastern Nepal it was 72.7% and 67.2% respectively, which is more as compared to our study.^8,12^

Among our study population, the majority of infants were breastfed 8-12 times a day 179 (58.69%) while 76 (24.92%) of them were breastfed 4-8 times per day, 31 (10.16%) breastfed >12 times a day and only 12 (3.93%) breastfed 4 times per day. In one breastfeeding practice study among US mothers, the average frequency of breastfeeding was 8 times per day at 1 month of age which decreased with an increase in age to 3.5 times per day at 1 year of age.^13^ In another study conducted in Iran, the frequency of breastfeeding was observed to be higher than the developing countries.^14^ Similar studies conducted in a rural area of Bangladesh revealed the greater frequency of breastfeeding among housewives compared to working mothers.^15^

In our study, 37 (12.13%) of the infants weighed below 2500 grams at birth. According to a study done in Africa, neonates with low birth weight (<2.5 kg) were more likely to not begin breastfeeding on demand than full-weight neonates.^16^ With the introduction of commercial milk formula in this market-driven world, early cessation of breastfeeding and discarding colostrum is still prevalent. So, necessary efforts should be made in explaining to the mothers and families the importance of breastfeeding and colostrum.^17^ Studies have suggested that maternal education, working status, infant's birth order, birth weight, maturity, antenatal counselling, and mode of delivery influences the practice of breastfeeding.^11^

As we conducted a descriptive cross-sectional study in a single tertiary care centre, our result might not be generalized in other settings and is different as compared to other studies conducted in different demographic regions of Nepal. Also, inferential statistics like association, and correlation could not be established from this study due to the limitation of the study design.

## CONCLUSIONS

The prevalence of colostrum feeding among newborns was higher as compared to other studies done in similar settings. However, proper counselling should be given to mothers and families regarding the importance of colostrum and breastmilk.
